# Pulmonary metastasis originating from colonic mucinous adenocarcinoma with a tree-in-bud pattern on computed tomography: A case report

**DOI:** 10.1016/j.rmcr.2025.102192

**Published:** 2025-03-10

**Authors:** Kensuke Takei, Hiroki Mori, Katsuyuki Asai

**Affiliations:** a General Thoracic Surgery, Hamamatsu Medical Center, Shizuoka, Japan; b Department of Pathology, Hamamatsu Medical Center, Shizuoka, Japan

**Keywords:** Computed tomography, Lung imaging, Tree-in-bud pattern, Pulmonary metastasis, Metastatic cancer, Colonic neoplasms

## Abstract

The tree-in-bud pattern, a chest computed tomography (CT) finding, is occasionally associated with malignant tumors. We report a surgical case of pulmonary metastasis from colonic mucinous adenocarcinoma presenting with a tree-in-bud pattern on chest CT. A 50-year-old man underwent surgery for descending colon cancer, which was histopathologically diagnosed as well-differentiated mucinous adenocarcinoma. During follow-up after adjuvant chemotherapy, chest CT revealed nodules with linear branching patterns in the left lung segments S9 and S10, consistent with a tree-in-bud pattern. Diagnostic thoracoscopic wedge resection of the left S9 was performed to exclude inflammatory disease, confirming metastatic colonic adenocarcinoma. Subsequently, 1 month later, left S10 segmentectomy was conducted. Histopathological examination revealed mucus filling the bronchioles in the affected area. This finding indicates that pulmonary metastases from colonic mucinous adenocarcinomas may present as a tree-in-bud pattern. CT findings of this pattern in patients with a history of colon cancer with extracellular mucus require thorough evaluation.

## Highlights

• The tree-in-bud pattern is a common chest CT finding.
• The tree-in-bud pattern is typically associated with inflammation.
• The tree-in-bud pattern is atypical for metastases from colon cancer.
• Extracellular mucus from mucinous adenocarcinoma caused this tree-in-bud pattern.

## 1 Introduction

The tree-in-bud pattern is a characteristic chest computed tomography (CT) finding comprising peripheral centrilobular nodules connected by linear and branching opacities, indicative of bronchiolar luminal impaction by pus, mucus, or fluid [1]. Typically, the tree-in-bud pattern is associated with peribronchiolar inflammation but has also been observed in malignancy [1,2]. Herein, we present a rare surgical case of pulmonary metastasis from colonic mucinous adenocarcinoma, presenting with a tree-in-bud pattern caused by extracellular mucus filling the affected bronchioles.

## 2 Case report

**Fig. 1:**
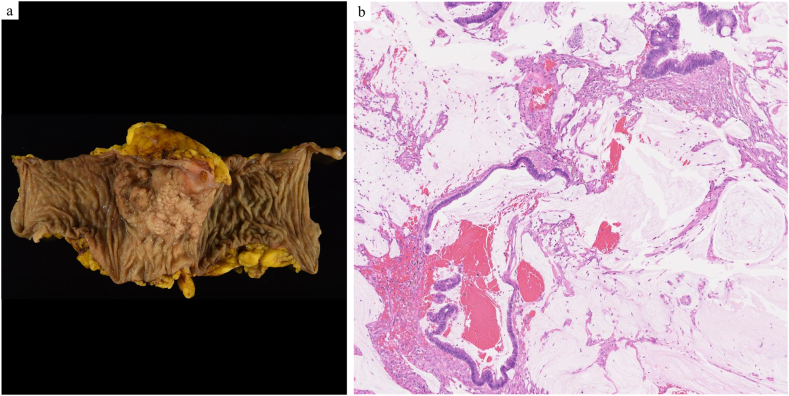
Pathological findings of colon cancer. a: Macroscopic finding of the descending colon. Type 1 tumor mass measuring 4.0 × 6.0 cm in diameter. b: Hematoxylin and eosin-stained image showing the tumor composed of papillary and tubular structures with columnar cells, accompanied by extracellular mucus (magnification × 5).

**Fig. 2:**
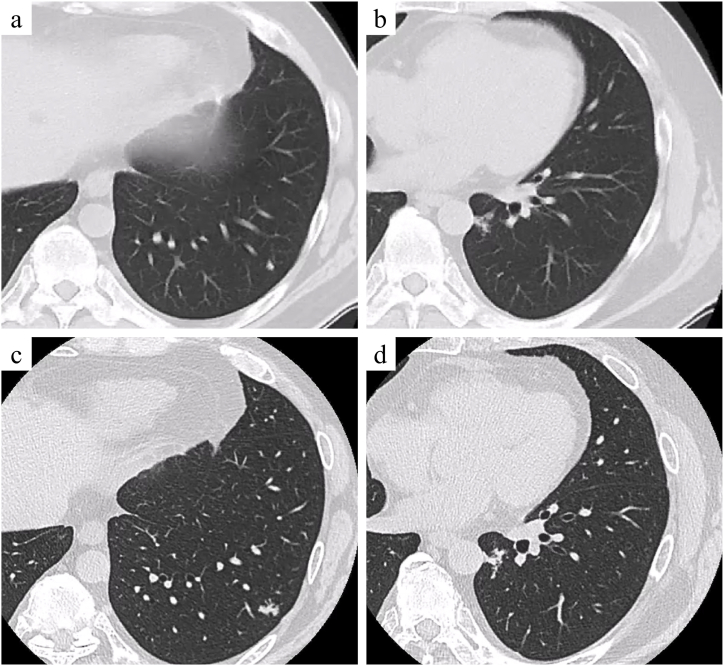
Radiological findings. a, b: Computed tomography showing nodules in segments S9 and S10 of the left lung, first detected after surgery for colon cancer (a: S9, b: S10). c, d: Nodules showing linear and branching opacities described as a tree-in-bud pattern, 15 months following initial nodule detection (c: S9, d: S10).

**Fig. 3:**
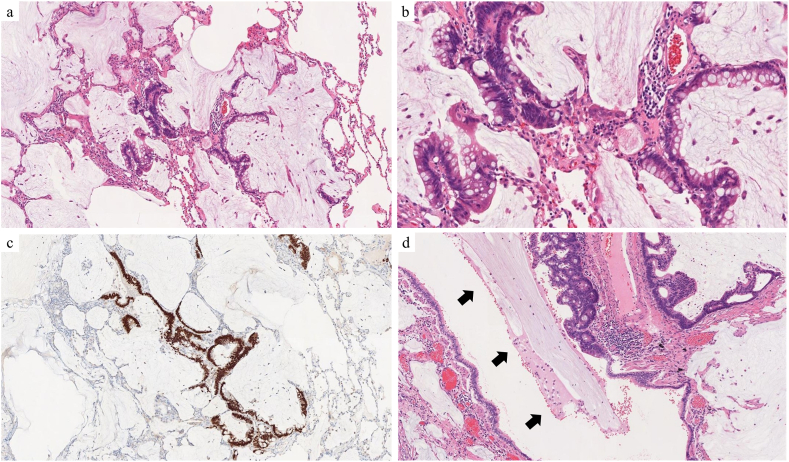
Pathological findings of pulmonary metastasis. a, b: Hematoxylin and eosin-stained image of the pulmonary tumor showing glandular and columnar cells with extracellular mucus (a: magnification × 4, b: magnification × 10). c: Immunohistochemical features indicate the expression of caudal-type homeobox transcription factor 2 (CDX2) (magnification × 4). d: Hematoxylin and eosin-stained image showing affected bronchioles filled with mucus (arrow; magnification × 4).

We report the case of a 50-year-old man who underwent surgery for colon cancer and later developed metachronous pulmonary metastases. The patient was a non-smoker with a history of asthma, managed using inhaled corticosteroids and long-acting β2-agonists. The patient underwent colectomy and D3 lymphadenectomy for descending colon cancer (Fig. 1a). Histopathological analysis confirmed a diagnosis of well-differentiated mucinous adenocarcinoma with pathological T3N2aM0 stage IIIB. The tumor exhibited papillary and tubular structures with columnar cells producing extracellular mucus (Fig. 1b). The patient completed eight cycles of adjuvant chemotherapy with capecitabine and oxaliplatin, followed by regular monitoring. At 14 months post-colectomy, chest CT revealed nodules in the S9 and S10 segments of the left lung (Fig. 2a and b). Over the subsequent 15 months, these nodules exhibited linear branching growth, described as a tree-in-bud pattern (Fig. 2c and d). No elevation in tumor markers, including carcinoembryonic antigen (CEA) and carbohydrate antigen 19-9 (CA19-9), was noted. Positron emission tomography-CT showed no lymph node involvement or distant extrathoracic metastases. These findings led to consideration of metastatic lung tumors from colon cancer or bronchiolar inflammation. A diagnostic thoracoscopic wedge resection of the left S9 segment was performed. Histopathological examination revealed a tumor comprising glandular and columnar cells with extracellular mucus (Fig. 3a and b). Immunohistochemistry demonstrated the expression of cytokeratin 20 (CK20) and caudal-type homeobox transcription factor 2 (CDX2) (Fig. 3c) with a lack of CK7, confirming pulmonary metastasis of colonic mucinous adenocarcinoma. At 1 month after S9 lung resection, left S10 segmentectomy was conducted. Histopathological examination confirmed pulmonary metastasis, with the bronchioles in the tumor area filled with mucus containing tumor cells (Fig. 3d).

## 3 Discussion

The tree-in-bud pattern is a common imaging finding in small airway diseases. Initially, this pattern was believed to signify the endobronchial spread of *Mycobacterium tuberculosis* [3]. However, subsequent reports identified other causes, including infectious diseases (bacterial, fungal, and viral), congenital disorders, immunologic conditions, and idiopathic disorders [1,2].

In this case, distinguishing between pulmonary metastasis and inflammation was crucial. Nodules in the left S9 and S10 segments exhibited the same tree-in-bud pattern on CT images. This pattern is commonly associated with inflammation. Considering the patient's history of inhaled corticosteroid use and adjuvant chemotherapy, inflammatory pulmonary disease was suspected. However, pulmonary metastasis from colon cancer could not be excluded. Both lesions were peripheral and inaccessible for bronchoscopic biopsy. Thoracoscopic surgery was performed to biopsy the left S9 lesions via wedge resection, confirming pulmonary metastasis from colon cancer. Several factors may explain the tree-in-bud pattern observed in pulmonary metastases from colon cancer.

Although typically linked to inflammation, this pattern has also been associated with malignancies. Pulmonary metastases can spread through hematogenous, lymphogenous, or aerogenous pathways. Tumor embolization in peripheral arteries, leading to vascular enlargement, may contribute to the tree-in-bud pattern [2,4]. Lymphogenous spread has also been associated with this pattern, as previously reported in pancreatic cancer metastases [5]. A rare case of pulmonary micropapillary adenocarcinoma exhibiting this pattern was attributed to micropapillary tufts floating in alveolar spaces [6]. Additionally, bronchial obstruction distal to malignant tumors may result in the tree-in-bud pattern. For instance, 22.5 % of patients with central lung cancer demonstrated the tree-in-bud pattern, predominantly due to mucoid impaction of distal bronchioles and bronchiolitis, with most cases involving squamous cell carcinoma [7].

The tree-in-bud pattern is atypical for colorectal cancer (CRC) metastases. Pulmonary metastases from CRC typically present as well-defined pulmonary nodules with smooth or lobulated margins on CT images [8]. In the present case with a tree-in-bud pattern, histopathological examination revealed the aerogenous spread of mucus containing tumor cells originating from colonic mucinous adenocarcinoma. Colorectal mucinous adenocarcinoma accounts for 10 %–15 % of CRC cases [9]. This subtype is defined by tumors with more than 50 % extracellular mucin content [10,11]. We believe that the observed tree-in-bud pattern resulted from extracellular mucus filling the bronchioles in the tumor-affected area. Pulmonary metastatic microangiopathy from colon cancer presenting as this pattern has been previously described [12]. However, to the best of our knowledge, there are no prior reports of pulmonary metastases from CRC involving the aerogenous spread of mucus containing tumor cells, presenting with a tree-in-bud pattern. Future case reports and series are necessary to better understand the mechanisms and clinical features underlying this finding in colorectal mucinous adenocarcinoma with pulmonary metastases.

## 4 Conclusion

This report describes a surgical case of pulmonary metastasis from colonic mucinous adenocarcinoma exhibiting a tree-in-bud pattern caused by extracellular mucus filling the bronchioles in the affected area. Our case highlights the importance of thoroughly evaluating pulmonary lesions in colon cancer patients with extracellular mucus during follow-up.

## CRediT authorship contribution statement

**Kensuke Takei:** Conceptualization, Data curation, Formal analysis, Investigation, Methodology, Writing – original draft. **Hiroki Mori:** Data curation, Formal analysis, Writing – review & editing. **Katsuyuki Asai:** Data curation, Formal analysis, Investigation, Methodology, Supervision, Writing – review & editing.

## Consent for publication

Consent for the publication of this case report was obtained.

## Funding

This research did not receive any specific grant from funding agencies in the public, commercial, or not-for-profit sectors.

## Declaration of competing interest

The authors declare that they have no known competing financial interests or personal relationships that could have appeared to influence the work reported in this paper.
